# Dual-Specificity Phosphatase 14 Regulates Zebrafish Hair Cell Formation Through Activation of p38 Signaling Pathway

**DOI:** 10.3389/fncel.2022.840143

**Published:** 2022-03-23

**Authors:** Guanyun Wei, Xu Zhang, Chengyun Cai, Jiajing Sheng, Mengting Xu, Cheng Wang, Qiuxiang Gu, Chao Guo, Fangyi Chen, Dong Liu, Fuping Qian

**Affiliations:** ^1^Key Laboratory of Neuroregeneration of MOE, Nantong Laboratory of Development and Diseases, School of Life Sciences, Co-innovation Center of Neuroregeneration, Nantong University, Nantong, China; ^2^Translational Medical Research Center, Wuxi No. 2 People’s Hospital, Affiliated Wuxi Clinical College of Nantong University, Wuxi, China; ^3^Department of Biomedical Engineering, Southern University of Science and Technology, Shenzhen, China; ^4^Department of Biology, Brain Research Center, Southern University of Science and Technology, Shenzhen, China

**Keywords:** DUSP14, supporting cell, hair cell, zebrafish, proliferation, regeneration

## Abstract

Most cases of acquired hearing loss are due to degeneration and subsequent loss of cochlear hair cells. Whereas mammalian hair cells are not replaced when lost, in zebrafish, they constantly renew and regenerate after injury. However, the molecular mechanism among this difference remains unknown. Dual-specificity phosphatase 14 (DUSP14) is an important negative modulator of mitogen-activated protein kinase (MAPK) signaling pathways. Our study was to investigate the effects of DUSP14 on supporting cell development and hair cell regeneration and explore the potential mechanism. Our results showed that *dusp14* gene is highly expressed in zebrafish developing neuromasts and otic vesicles. Behavior analysis showed that *dusp14* deficiency resulted in hearing defects in zebrafish larvae, which were reversed by *dusp14* mRNA treatment. Moreover, knockdown of *dusp14* gene caused a significant decrease in the number of neuromasts and hair cells in both neuromast and otic vesicle, mainly due to the inhibition of the proliferation of supporting cells, which results in a decrease in the number of supporting cells and ultimately in the regeneration of hair cells. We further found significant changes in a series of MAPK pathway genes through transcriptome sequencing analysis of *dusp14*-deficient zebrafish, especially *mapk12b* gene in p38 signaling. Additionally, inhibiting p38 signaling effectively rescued all phenotypes caused by *dusp14* deficiency, including hair cell and supporting cell reduction. These results suggest that DUSP14 might be a key gene to regulate supporting cell development and hair cell regeneration and is a potential target for the treatment of hearing loss.

## Introduction

Millions of people all over the world are subjected to different degrees of hearing loss (Morton, 1991; Sun, 2021). The main causes of hearing impairment include aging, noise, genetic mutations, and exposure to ototoxic drugs, which contributed to the sensory hair cell loss in inner ear (Furness, 2015). Most cases of acquired hearing loss are due to degeneration and subsequent loss of cochlear hair cells (Vlajkovic and Thorne, 2021). However, there are no Food and Drug Administration (FDA)-approved drugs to protect from hearing loss, which makes urgent the task of discovering new therapeutics.

The auditory epithelium located in the cochlea is composed of sensory hair cells and non-sensory supporting cells (Kelley, 2006). Although hair cells cannot regenerate in mammalian adults, regeneration of hair cells in the inner ear and lateral line is widespread in non-mammalian vertebrates, such as chicken, amphibian, and zebrafish. Additionally, those hair cells regenerate from transdifferentiating supporting cells, which aroused the interest of many auditory scientists in understanding the inner ear hair cell and supporting cell development and regeneration, with the potential to develop biologic therapies for hearing loss (Rubel et al., 2013). Therefore, it is particularly important to understand the cellular and molecular mechanisms of such striking difference between mammalian and non-mammalian vertebrates.

Dual-specificity phosphatase (DUSP) family is a group of phosphatases, which dephosphorylate of tyrosine and/or serine or threonine residues and the resulting activity regulation of their substrates (Caunt and Keyse, 2013). DUSPs are considered to be major modulators of key signaling pathways that are dysregulated in a variety of diseases (Patterson et al., 2009a; Ríos et al., 2014; An et al., 2021). DUSPs can be divided into seven subgroups with the consideration of substrate preferences: phosphatases of regenerating liver (PRL) family, cell division cycle 14 (CDC14) phosphatases, phosphatase and tensin homologs deleted on chromosome 10 (PTEN), slingshot homolog (SSH) family of phosphatases, myotubularins, mitogen-activated protein kinase phosphatases (MKPs), and atypical DUSPs (Huang and Tan, 2012). Atypical DUSPs are mostly with low molecular weight and lack the N-terminal two CDC25 homology 2 (CH2) domain. The most atypical DUSPs are localized in the cytoplasm. It is reported that some atypical DUSPs regulate MAPK, including extracellular signal-regulated kinases (ERKs), c-Jun N-terminal kinases (JNKs), and p38 kinases, which plays an important role in cell proliferation and apoptosis (Huang and Tan, 2012).

Dual-specificity phosphatase 14, an atypical member of the DUSP family of proteins, are critical modulators in various biological processes, such as apoptosis, inflammation, proliferation, and oxidative stress. Our results showed that *dusp14* gene was expressed in the inner ear and lateral line system of zebrafish, which suggest that Dusp14 may play a vital role in the regulation of hair cell fate in zebrafish.

Based on these findings, this study examined whether *dusp14* gene could regulate the hair cell fate in zebrafish, especially the behavior of supporting cell. Furthermore, we aimed to evaluate whether *dusp14* gene putative modulating hair cell actions would be related to modulation of the MAPK signaling pathway.

## Materials and Methods

### Zebrafish Embryos

Zebrafish (*Danio rerio*) were reared and maintained at 28.5°C as Westerfield described (Westerfield, 1995). Two zebrafish lines were used in the study, including the wild-type AB line and the transgenic line *Tg(Brn3c:mGFP)*, where membrane-localized green fluorescent protein (GFP) is specifically expressed in the hair cells. Embryonic stages are defined as described (Kimmel et al., 1995). Embryos were collected after natural spawns. Embryos were moved to embryo medium containing 0.2 mM phenylthiourea at ∼20 h postfertilization (hpf) to prevent pigmentation. All animal procedures were performed according to protocols approved by the Animal Care and Use Committee of Nantong University and were consistent with the National Institutes of Health Guide for the Care and Use of Laboratory Animals.

### Whole-Mount *in situ* Hybridization

The DNA fragments of zebrafish *dusp14* and *eya1* were amplified by PCR using the primers (Supplementary Table 3). Then, they were subcloned into the pGEM-T Easy Vector (Promega, United States), and a gene-specific digoxigenin-labeled RNA probe was transcribed *in vitro* using the DIG RNA Labeling Kit (SP6&T7) (Roche) following the manufacturer’s instructions. The prefixed embryos were incubated with the probe overnight at 4°C. The alkaline phosphatase (AP)-conjugated antibody against digoxigenin (Roche) was used to detect the digoxigenin-labeled RNA probe. The AP-substrate NBT/BCIP solution (Roche) was added to the reaction system to stain the tissues specifically expressing *dusp14* gene or *eya1* gene in zebrafish.

### Morpholino and CRISPR/Cas9 Microinjection

According to the manufacturer’s instruction, morpholino antisense oligos (MOs; Gene Tools) were prepared at a stock concentration of 1 mM. We designed *dusp14* splice-modifying morpholino (*dusp14*-MO) to knockdown the expression of *dusp14* (Supplementary Table 3). MOs were diluted to 0.3 mM and injected into one-cell-stage embryos.

To generate the *dusp14* gene mutant zebrafish, as described in our previous work (Gong et al., 2017), 2–3 nL of solution containing specific single-guide RNA (sgRNA) and Cas9 mRNA was injected into one-cell-stage embryos (primers used are listed in Supplementary Table 3).

### mRNA Synthesis and Phenotypic Rescue

*dusp14* DNA fragments were synthesized by PCR using the primers, *dusp14-Bam*HI-F and *dusp14-Eco*RI-R (Supplementary Table 3). The DNA fragments were subcloned into the pCS2 + vector, and the recombinant plasmid was linearized using the restriction endonuclease *Not*I (New England Biolabs). The linearized product was purified as a template and transcribed into mRNA *in vitro* using the mMESSAGE mMACHINE High Yield Capped RNA Transcription kit (Ambion). Then, the synthesized *dusp14* mRNA was coinjected with *dusp14*-MO into one-cell-stage zebrafish embryos. The rate of rescued zebrafish after injection of *dusp14* mRNA was analyzed at 72 hpf.

### RNA Extraction, Reverse Transcription, and Quantitative Real-Time PCR

Total RNAs of all samples were extracted using TRIzol reagent (Invitrogen, United States), and genomic DNA contamination was removed by DNase I (Promega, United States). The RNA yield was determined using NanoDrop ND-2000 (Thermo Fisher Scientific, United States), and integrity was checked on a 1% agarose gel. The cDNA was synthesized using oligo-dT primers and Superscript RT-III (Takara, JP). Quantitative real-time PCR (qRT-PCR) was performed using a Plus Real-Time PCR System (Applied Biosystems, United States). SYBR Prime ScriptTM RTPCR kit (Takara, JP) was used for mRNA qRT-PCR. Data were analyzed using the relative 2^–ΔΔ*CT*^ method (Livak and Schmittgen, 2001; Schmittgen and Livak, 2008). The primers for qRT-PCR are listed in Supplementary Table 3.

### Acoustic or Vibrational Startle

Acoustic or vibrational startle protocol was based on previous studies (van den Bos et al., 2017) with appropriate modifications. Larvae (6 dpf) were transferred from petri dishes to wells filled with 1 mL of E3 medium. Both the control and *dusp14*-MO zebrafish were tested. The protocol (lights on) consisted of 10-min acclimation, followed by 9 acoustic or vibrational stimuli (DanioVision intensity setting) with a 20-s interstimulus interval (ISI). Variable of interest to show the startle response was maximum velocity (mm/s) with 1-s intervals, since the startle response is a short burst of activity best captured by this parameter. When subjects did not show a clear response to the first stimulus (values lower than 15 mm/s), they were discarded from analysis.

### Acoustic Startle Reflex

The acoustic startle reflex was performed as described previously (Yang et al., 2017). The larvae (5 dpf) were put in a thin layer of culture media in a petri dish attached to mini vibrator. The response of larvae to sound stimulus (a tone burst 9 dB re. m s^–2^, 600 Hz, for 30 ms) generated by the vibrator was recorded from above by an infrared camera over a 6-s period. The mean moving distance and peak speed were used to quantify the startle response.

### Immunofluorescence Staining

For immunofluorescence staining, the embryos were anesthetized and then fixed using 4% paraformaldehyde. After washing three times with PBS-T, the embryos were incubated in the antigen retrieval solution (Beyotime Biotechnology, China, #P0088) for 15 min at 98°C. Non-specific binding was then blocked with 10% donkey serum (Solarbio, China, #SL050) in PBS-T. Next, specific primary antibodies against GFP (Abcam, #ab13970) and cleaved caspase-3 (CST, #9664), 5-bromo-2′-deoxyuridine (BrdU) (Sigma, #B5002) or SOX2 (Abcam, #ab97959) were added, and secondary antibodies were used to detect the primary antibodies.

TdT-mediated dUTP nick end labeling (TUNEL) assay was performed according to the manufacturer’s instructions (Alexa Fluor 640, cat#: 40308ES20, YEASEN Biotech Co. Ltd) to detect cell death in the HCs of neuromast. In brief, the embryos were anesthetized and then fixed using 4% paraformaldehyde. After washing three times with PBST, 20 μg/mL proteinase K (Roche) was used to treat the embryos. Next, Alexa Fluor 640-12-dUTP Labeling Mix was applied to label the apoptotic cells for at least 3 h. DAPI was applied to label the nucleus.

Images were taken with a Nikon confocal microscope A1R at 40× magnification and were analyzed by Nikon A1R NIS Elements. Exposure settings were adjusted to minimize oversaturation.

### Drug Treatment

The p38 inhibitor (APExBIO, #C5248), with a working concentration at 50 ng/μL, was coinjected with *dusp14*-MO into the 1–2 cell-stage zebrafish embryos. Additionally, the injected zebrafish were raised at 28.5°C. The development status was recorded with a bright field microscope at about 72 hpf.

### Statistical Analysis

All data were analyzed using GraphPad Prism 8.3.0. One-way ANOVA, unpaired Student’s *t*-tests, and two-way ANOVA were used to determine statistical significance when comparing two groups. The value of *p* < 0.05 was considered as statistically significant. All data are presented as means with SEM, and all experiments were repeated at least three times.

### RNA-Seq Analysis

To study gene expression changes after *dusp14* knockdown in zebrafish, we performed transcriptome sequencing. The wild-type zebrafish and *dusp14-*MO injected zebrafish of 3 days old were prepared, respectively. Three independent replicates of the samples were analyzed for each treatment. The total RNA was extracted using TRIzol reagent (Invitrogen, United States) following the manufacturer’s procedure. The quantity and purity of total RNAs were checked using a Bioanalyzer 2100 and RNA 6000 Nano LabChip Kit (Agilent, CA, United States) with RIN value > 7.0. All RNA samples were submitted to GENEWIZ Science (Suzhou, China), and deep sequencings were performed on an Illumina Hiseq2500.

For RNA-seq data, the Cuffdiff was used to estimate differential expression between samples at the transcript level (Kim et al., 2013). The differentially expressed gene (DEG) was determined with *log2 fold change* > 1, *p-value* < 0.05. The R package Clusterprofer was used for Kyoto Encyclopedia of Genes and Genomes (KEGG) pathway and gene ontology (GO) annotation.

## Results

### The *dusp14* Gene Is Expressed in the Otic Vesicles and Neuromasts in Zebrafish

**FIGURE 1:**
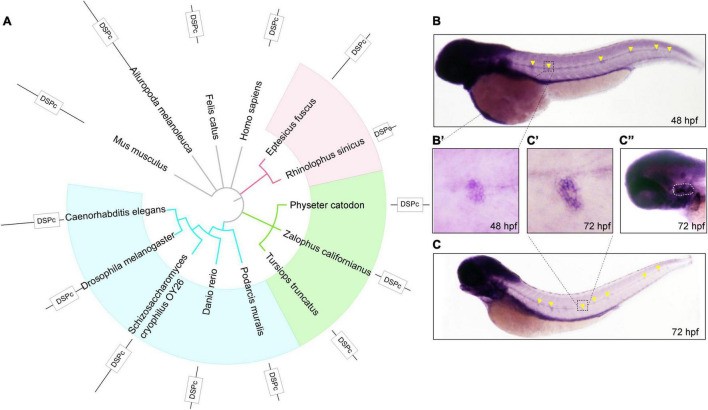
The phylogenetic and expression analysis of zebrafish *dusp14*. **(A)** A phylogenetic tree was generated using the PhyML software base on amino acid sequences. The pictures are mouse, giant panda, domestic cat, human, big brown bat, Chinese horseshoe bat, sperm whale, sea lion, bottlenose dolphins, lizards, zebrafish, Saccharomyces cerevisiae, and nematodes. **(B,C)** WISH with the *dusp14* probe in wild-type zebrafish showed that the gene is expressed in the otic vesicle and lateral line at 48 hpf **(B)** and 72 hpf **(C)**. **(B’,C’,C”)** Are the detailed information with higher magnification of the **(B,C)**, respectively.

First, to observe the conservation of DUSP14, we constructed an evolutionary tree of 14 species based on the amino acid sequence of DUSP14, including mouse, giant panda, domestic cat, human, big brown bat, Chinese horseshoe bat, sperm whale, sea lion, bottlenose dolphins, lizards, zebrafish, yeast, and nematodes (Figure 1A). The results showed that *dusp14* is an extensive conserved gene in many species with DUSP (dual-specificity phosphatases) conserved domain.

Next, to investigate the spatiotemporal expression of *dusp14* during the embryonic development, we performed the WISH using a *dusp14* antisense probe. As shown in Figures 1B,B′, *dusp14* is expressed in the neuromast at 48 hpf. At 72 h (Figure 1C), the *dusp14* gene expression is pronounced in the lateral line (Figure 1C′) and the otic vesicle (Figure 1C′′). These results indicate that *dusp14* may play an important role in inner ear development.

### Knockdown the *dusp14* Expression by Morpholino Leads to Hearing Defects in Zebrafish

**FIGURE 2:**
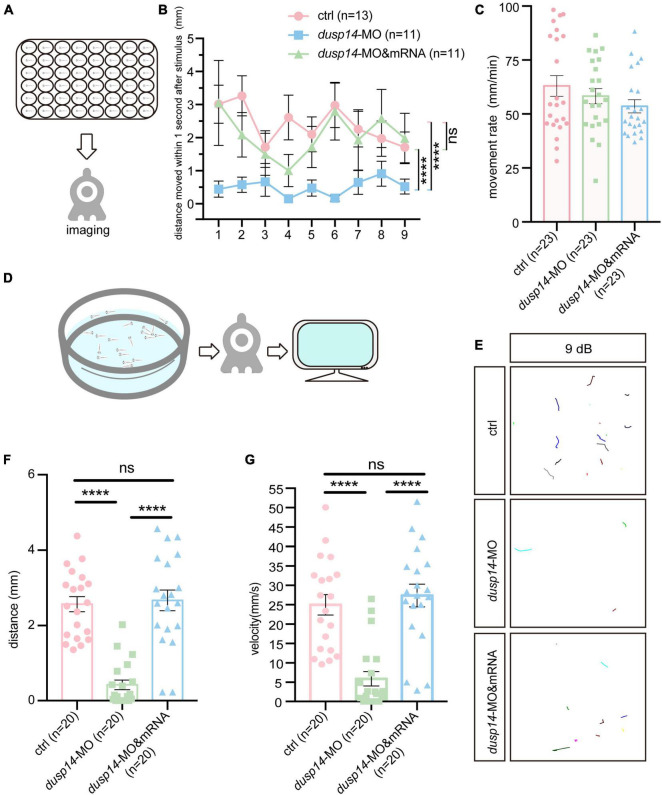
Knockdown of *dusp14* gene affects the hearing-related behavioral response in zebrafish. **(A)** Working diagram of acoustic or vibrational startle test after *dusp14*-MO injection at 5 dpf. **(B)** The distance moved of zebrafish within 1 s after the percussion stimulation. **(C)** Statistics of the moving rate of zebrafish during the total test. **(D)** The schematic diagram shows the startle response testing equipment. **(E)** The swimming trajectory of the control, *dusp14* morphants and *dusp14*-mRNA and MO. **(F,G)** Swimming distance and peak velocity of zebrafish larvae at 5 dpf that reflected the auditory function of zebrafish larvae by examining the startle response. Values with **** above the bars are significantly different (*P* < 0.0001), ns means no significance.

To explore the effect of the *dusp14* gene on zebrafish behavioral response, we designed morpholino oligonucleotides to knockdown the expression of *dusp14*, and *dusp14*-MO was validated to effectively reduce *dusp14* expression (Supplementary Figure 1). Then, we performed acoustic or vibrational startle test after *dusp14*-MO injection at 5 dpf. The results showed that the response of *dusp14*-MO zebrafish to percussion stimuli becomes sluggish, whereas the injection of *dusp14* mRNA significantly rescued the phenotype (Figures 2A,B). In addition, there is no difference in movement rate among wide-type zebrafish, *dusp14*-MO zebrafish, and *dusp14*-mRNA and MO coinjected zebrafish during the total test (Figure 2C), which suggests that the injection did not affect locomotor activities of zebrafish.

To further investigate the effects of *dusp14*-MO on hearing dysfunction, we performed the acoustic startle reflex experiment (Figure 2D). The results showed that the swimming trajectory (Figure 2E), swimming distance (Figure 2F), and velocity (Figure 2G) of *dusp14* morphants were reduced compared to the controls, which were reversed by *dusp14*-mRNA. These results indicate that *dusp14* is necessary for hearing-related behaviors.

### Knockdown *dusp14* Expression by Morpholino Decreases the Number of Hair Cells and Neuromasts in Zebrafish

**FIGURE 3:**
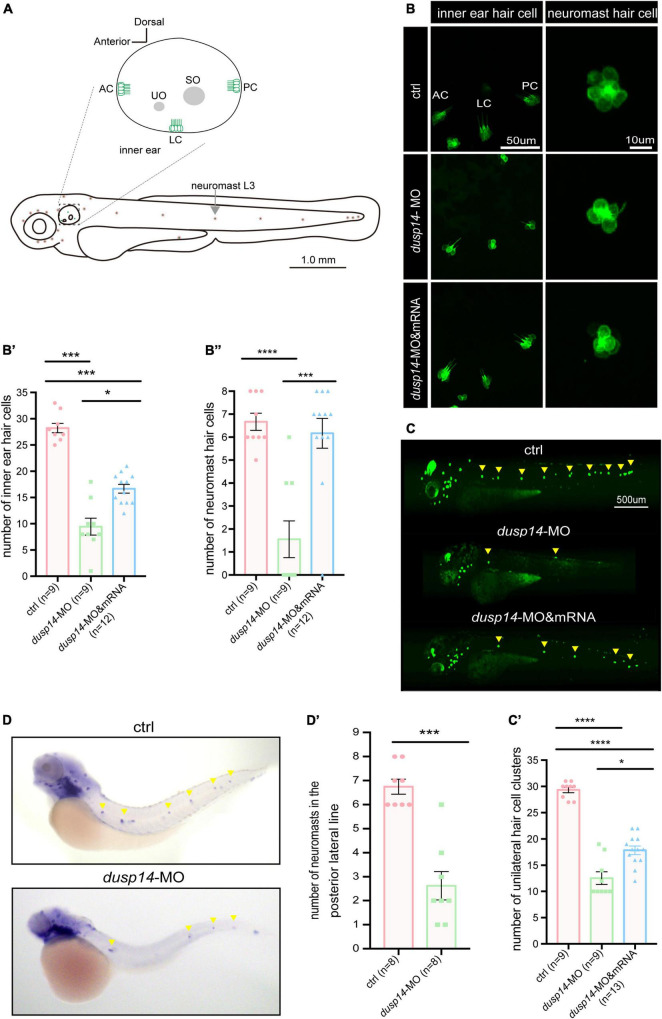
Knockdown *dusp14* expression by morpholino decreases the number of hair cell and neuromasts in zebrafish. **(A)** Schematic diagram of zebrafish neuromast and inner ear hair cell. **(B)** Confocal imaging analysis of crista hair cells in the otic vesicle of control and *dusp14* deficiency zebrafish at 72 hpf. **(B′)** The statistical analysis of the numbers of inner ear crista hair cells in the control and *dusp14* morphants at 72 hpf. **(B′′)** The statistical analysis of the numbers of hair cells in the remaining neuromast L3 in the control and *dusp14* morphants at 72 hpf. **(C)** The imaging analysis of control and *dusp14* morphants at 72 hpf in fluorescent field. Scale bar = 500 μm. **(C′)** Quantification of the number of unilateral hair cell clusters of control and *dusp14* morphants at 72 hpf. **(D)** *in situ* hybridization of the *eya1* gene specifically expressed in the neuromasts showed that the number of neuromasts in *dusp14*-MO zebrafish was decreased. **(D′)** statistics results of D. Each bar represents the mean ± SEM. Values with *, ***, and **** above the bars are significantly different (*P* < 0.05, *P* < 0.001, and *P* < 0.0001, respectively).

It is well known that hair cells in the inner ear of zebrafish are responsible for the balance perception and hearing. Therefore, to test the underlying cellular mechanisms of *dusp14* gene on ear function, we knockdown the *dusp14* expression by morpholino in the transgenic zebrafish line *Tg*(*Brn3c:mGFP*), in which the GFP were specifically expressed in hair cells (Figure 3A). The results showed that the number of hair cells in three different crista hair cell clusters (anterior crista hair cells: AC; lateral crista hair cells: LC; posterior crista hair cells: PC) was reduced after *dusp14-*MO microinjection at 72 hpf (Figures 3B,B′), which was neutralized by *dusp14* mRNA treatment.

The hair cells in zebrafish are also present in the lateral line system containing the neuromasts that is important to perceive changes in the surroundings. Then, we further investigate the role of *dusp14* gene on neuromast formation and found that the number of hair cell clusters in the posterior lateral line of *dusp14* morphants was significantly decreased at 72 hpf (Figures 3C,C′). Moreover, the number of the hair cells in the remaining neuromast L3 was also decreased (Figures 3B,B′′). But the *dusp14* mRNA treatment interfered these changes remarkably. In addition, we used the *eya1* gene to label the neuromast cells (Grant et al., 2005; Whitfield, 2005) in lateral line by WISH and discovered that the number of neuromasts in the posterior lateral line of *dusp14* morphants was significantly reduced (Figures 3D,D′). These results indicate that *dusp14* gene affects the number of hair cells in zebrafish inner ear and lateral line.

To further validate the function of *dusp14* during the hair cell development, the CRISPR/Cas9 system was utilized to knockout *dusp14* in wild-type zebrafish. As shown in Supplementary Figure 2A, we chose a sgRNA target site near the translation start codon in the exon1 of *dusp14* for CRISPR/Cas9-mediated mutation to abolish the protein translation. Similar to the results of the *dusp14* morphants, the number of hair cell clusters and hair cells in the posterior lateral line of the *dusp14* mutants was remarkably fewer than that of the control fish at 72 hpf, which was partially reversed by the *dusp14* mRNA injection (Supplementary Figures 2C,C′,C′′).

### Knockdown of the *dusp14* Gene Reduces the Number of Supporting Cells and Proliferation of Supporting Cells

As we know, hair cells of the inner ear and lateral line system in zebrafish regenerate from mitotic supporting cells (Williams and Holder, 2000; Kniss et al., 2016; Thomas and Raible, 2019). Since knockdown of the *dusp14* gene caused the reduction of hair cells, we speculated that support cells, resource of hair cells, were also affected by *dusp14* morpholino injection.

**FIGURE 4:**
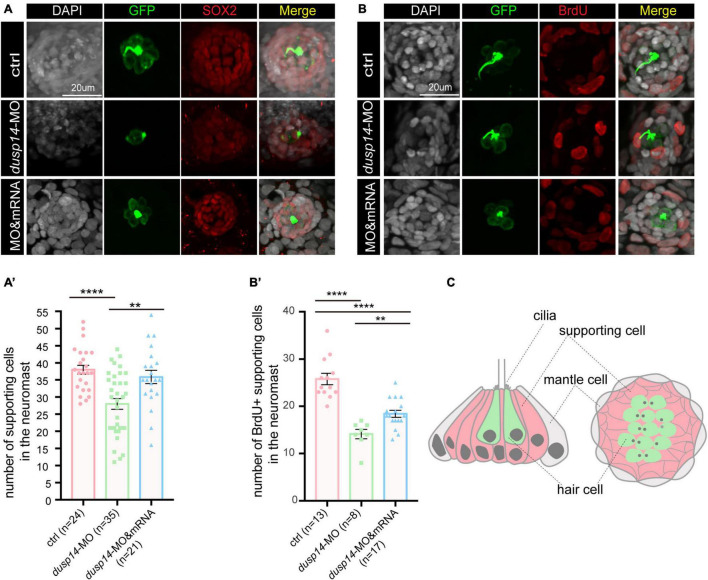
Knockdown of the dusp14 gene reduces the number of supporting cells and proliferation of supporting cells. **(A)** The representation of SOX2 immunofluorescence images of neuromasts in the posterior lateral line of the control and *dusp14* morphants. **(A′)** Quantification of the number of supporting cells in the posterior lateral line neuromast of control and *dusp14* mutants at 72 hpf. **(B)** BrdU staining for the supporting cell in the neuromasts in the posterior lateral line of the control zebrafish and *dusp14* morphants. Scale bar = 20 μm. **(B′)** Quantification of zebrafish embryos with the BrdU^+^ cells in the control and *dusp14* morphants. **(C)** Schematic diagram of the longitudinal structure and the plane structure of neuromast, the gray part is the mantle cell, the pink part is supporting cell, and the green represents hair cell. Experimental embryos were sampled at 72 hpf (*n* > 8). Each bar represents the mean ± SD. Values with ** and **** above the bars are significantly different (*P* < 0.01 and *P* < 0.0001, respectively).

To test the hypothesis, we analyzed SOX2^+^ cells in the posterior lateral line of *dusp14* morphants by immunostaining with anti-SOX2 antibody. The results showed that SOX2^+^ cell number was dramatically decreased after *dusp14* morpholino injection, which was substantially reserved by *dusp14* mRNA treatment (Figures 4A,A′). Furthermore, we performed the BrdU incorporation to measure the regeneration of supporting cells in the lateral line (Figures 4B,B′). Our results showed that the number of proliferating supporting cells was significantly reduced in the *dusp14* morphants. Additionally, the *dusp14* mRNA treatment partially interfered these changes. What is more, there is no difference in apoptotic signal between *dusp14* morphants and the control, which was immunostained with TUNEL and cleaved caspase 3 in zebrafish neuromasts (Supplementary Figure 3). Taken together, we found that *dusp14* gene may regulate the formation of hair cells and ultimately affect the hearing by modulating the proliferation of supporting cells in the zebrafish lateral line (Figure 4C).

### Transcriptomic Sequencing Data Revealed p38 Signaling Pathways May Responsible for Regulation of *dusp14* Gene on Hearing Function

**FIGURE 5:**
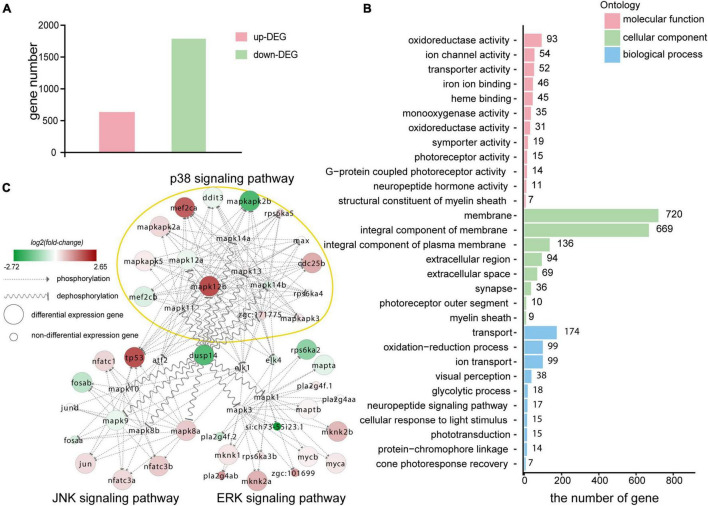
Transcriptomic sequencing data revealed p38 signaling pathways may responsible for regulation of dusp14 gene on hearing function. **(A)** The upregulated differentially expressed genes (DEGs) and downregulated DEGs that might be affected by knockdown *dusp14* based on transcriptome data analysis. **(B)** Gene ontology (GO) annotation enrichment of DEGs. **(C)** Gene expression changes in the MAPK pathway caused by *dusp14* knockdown.

To gain a further insight into the molecular mechanism by which *dusp14* gene responsible for the regeneration of the supporting cells, we performed RNA sequencing (RNA-seq) of 72 hpf wild-type zebrafish and *dusp14*-MO zebrafish. Over 40 million valid reads per library on average were obtained after quality filtering, which was, respectively, mapped to about 90% of the zebrafish genome (Supplementary Table 1). The results revealed 2,418 DEGs that might be affected by the absence of *dusp14* with 1,787 upregulated DEGs and 631 downregulated DEGs (Figure 5A and Supplementary Table 2). What is more, 12 genes (*apoeb, gsta.q, pkma, meis2b, dld, prkcbb, mag, ush1ga, mkm2os.2, fbxo16, pcloa, and cyp26c1*) were randomly selected for qRT-PCR analysis to confirm the quality of RNA-seq data. Notably, the expression changes of 6 among 12 genes were consistent with the results of RNA-seq data analysis (Supplementary Figures 4A,B), indicating the reliability of RNA-seq analysis.

Subsequently, we performed KEGG pathway enrichment analysis on the RNA-seq data. The results showed that knockdown *dusp14* gene by morpholino mainly resulted in changes in various metabolic pathways (Supplementary Table 2), including carbon metabolism, tryptophan metabolism, glycine metabolism, serine metabolism, tyrosine metabolism, retinol metabolism, metabolism of xenobiotics by cytochrome P450, phenylalanine metabolism, and so on. Furthermore, the most significant GO analysis enrichment terms were oxidoreductase activity, which were assigned as molecular functions. The cell membrane was the most significant GO enrichment term assigned as cellular component, and regulation of transportation was the most significant GO enrichment term assigned as a biological process (Figure 5B).

The DUSP family targets to MAPK signaling pathway, which modulate diverse cellular functions, such as regeneration, differentiation, and apoptosis. Therefore, we observed the gene expression changes in p38 kinases, ERKs, and JNKs signaling pathway in RNA-seq data. As is shown in Figure 5C, the genes directly regulated by *dusp14* dephosphorylation include *mapk8b*, *mapk9*, *mapk12a*, and *mapk12b*, which are mainly in the JNK and p38 signaling pathways. Among them, *mapk12b* belonging to p38 signaling pathway showed the most significant difference, which suggests that p38 signaling pathway may play a vital role in *dusp14* regulation of hearing in zebrafish.

### p38 Signaling Pathway Is Involved in *dusp14* Regulation of Inner Ear Hearing in Zebrafish

**FIGURE 6:**
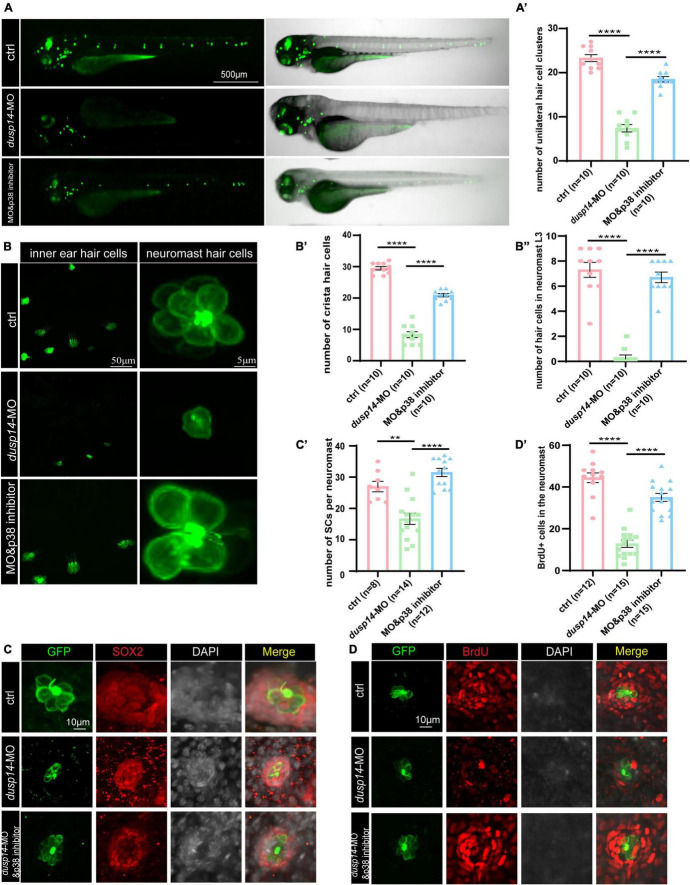
p38 signaling pathway is involved in dusp14 regulation of inner ear hearing in zebrafish. **(A)** The imaging analysis of control and *dusp14* mutants at 72 hpf in bright field and fluorescent field. **(A′)** Quantification of the number of the unilateral hair cell clusters of control and *dusp14* mutants at 72 hpf. **(B)** Confocal imaging analysis of inner ear hair cells and lateral line neuromast hair cells of control, *dusp14* deficiency and dusp14-MO and p38 inhibitor coinjected zebrafish at 72 hpf. **(B′)** The statistical analysis of the numbers of crista hair cells for panel **(B)**. **(B′′)** The statistical analysis of the numbers of hair cells in the remaining neuromast L3 for panel **(B)**. **(C)** The representation of SOX2 immunofluorescence images of neuromasts in the posterior lateral line of the control, *dusp14* morphants and dusp14-MO and p38 inhibitor coinjected zebrafish at 72 hpf. **(C′)** Quantification of the number of supporting cells per neuromast for panel **(C)**. **(D)** BrdU staining for the supporting cells in the neuromasts of the control, *dusp14* morphants and dusp14-MO and p38 inhibitor coinjected zebrafish at 72 hpf. **(D′)** Quantification of zebrafish embryos with the BrdU^+^ cells for panel **(D)**. Experimental embryos were sampled at 72 hpf (*n* > 6). Each bar represents the mean ± SD. Values with ** and **** above the bars are significantly different (*P* < 0.01 and *P* < 0.0001, respectively).

To further explore whether p38 signaling is responsible for *dusp14* regulation of hearing in zebrafish, the wild-type and *dusp14*-MO zebrafish were treated with p38 inhibitor. As previously shown, although knockdown *dusp14* gene expression by morpholino significantly decreased the number of hair cell clusters (Figures 6A,A’), the number of hair cells in inner ear cristae and neuromast L3 in the posterior lateral line, p38 inhibitor dramatically reversed these changes (Figures 6B,B’,B”). Additionally, p38 inhibitor also blocked the effect of *dusp14*-MO on the number of supporting cell and the proliferation of supporting cells (Figures 6C,C’,D,D’). These results indicate that p38 signaling pathway is necessary for the *dusp14* regulation of hearing.

## Discussion

**FIGURE 7:**
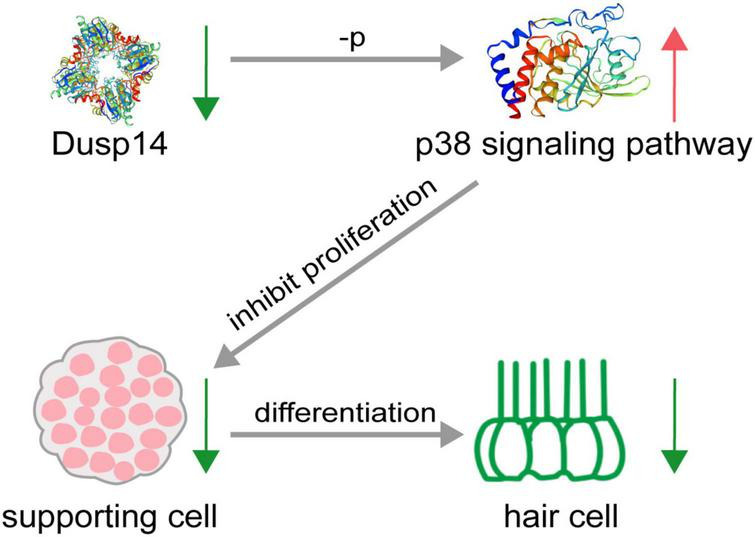
Schematic diagram of hair cell reduction caused by *dusp14* knockdown. Knockdown of *dusp14* expression by morpholino inhibited the proliferation of supporting cells by p38 signaling pathway, resulting in a decrease in the number of hair cells and ultimately leading to abnormal hearing and balance-related behaviors in zebrafish.

Hearing loss in mammals mainly caused by degeneration of hair cells in the inner ear, which is an irreversible process. To date, apart from hearing aids and cochlear implants, no pharmacological therapy promoting functional recovery from hearing loss is clinically available. This study demonstrates that *dusp14*, a conserved gene between species, is highly expressed in the lateral line and otic vesicles in zebrafish. Knockdown of *dusp14* expression by morpholino inhibited the proliferation of supporting cells through p38 signaling pathway, resulting in a decrease in the number of hair cells and ultimately leading to abnormal hearing and balance-related behaviors in zebrafish (Figure 7).

Dual-specificity phosphatase 14, a member of the atypical DUSPs, has been implicated in inflammation, apoptosis, cancer, diabetes, cell differentiation, and proliferation (Wada and Penninger, 2004; Turjanski et al., 2007; Lountos et al., 2009; Ríos et al., 2014). Dusp14 was characterized by yeast two hybrid systems for the first time to identify a new protein interacting with T cell costimulatory CD28 (Marti et al., 2001). It is reported that DUSP14 knockout aggravated pathological processes involved in non-alcoholic fatty liver disease development, whereas DUSP14 overexpression ameliorated these pathological alterations (Wang S. et al., 2018). Moreover, hepatic ischemia–reperfusion injury reduced Dusp14 expression, which suggests that Dusp14 is a protective factor in liver damage (Wang X. et al., 2018). Suppressing DUSP14 expression exacerbated cardiac injury through activating MAPK signaling pathways (Lin et al., 2018). Cardiac-specific Dusp14 overexpression alleviated aortic banding-induced cardiac dysfunction and remodeling (Li et al., 2016). These results indicate that DUSP14 may be a positive regulator of various cellular responses. We found that *dusp14* gene is important to supporting cell proliferating in zebrafish. Highly *dusp14* expression in the lateral line and otic vesicle in zebrafish was observed, however, whether that *dusp14* gene is specific located in the supporting cell remains unclear. The more experiments are needed.

*Dusp14* is a dephosphorylate regulator that mainly acts on MAPK signaling and has different MAPK pathway targets in the pathological process of various diseases. *In vitro*, GST-tagged DUSP14 can dephosphorylate p38, ERK, and JNK pathways (Patterson et al., 2009b). DUSP14 knockout mice after myocardial ischemia–reperfusion injury induce the activation of MAPKs, including elevated p-p38, p-ERK1/2, and p-JNK in heart tissues (Lin et al., 2018). However, Dusp14 deficiency after hepatic ischemia–reperfusion injury upregulated p-JNK1/2 and p-p38, but not p-ERK1/2 (Wang X. et al., 2018). The enhanced phosphorylated p38 and JNK1/2 levels, but not ERK1/2, were also observed in Dusp14 knockout mice in aortic banding-induced hypertrophic heart tissues (Wang X. et al., 2018). The phosphorylation of ERK and JNK but not p38 increased in dominant-negative Dusp14-transduced primary T cells (Galvao et al., 2018). DUSP14 negatively regulated ERK1/2 pathway in T-cell proliferation (Sun et al., 2021). Our transcriptomic sequencing analysis showed that genes mainly regulated by *dusp14* dephosphorylation belong to the JNK and p38 signaling pathways in *dusp14*-MO zebrafish. Additionally, p38 signaling, *mapk12b* gene, increased mostly in morpholino-induced *dusp14*-deficient zebrafish. In addition, p38 inhibitor significantly inhibited the effect of *dusp14*-MO on the proliferation of supporting cells and the decrease of hair cell in zebrafish. However, further experiments are needed to confirm the regulation of *mapk12b* gene on zebrafish supporting cells. Whether JNK and ERK are involved in regulating supporting cell development and how important their roles are also needed to be further investigated.

Numerous studies in non-mammalian species since the initial discoveries have elucidated that inner ear hair cell regeneration happens by two methods: supporting cells directly transdifferentiate into hair cells, supporting cells mitosis and then one of the daughter cells transdifferentiate into hair cells (Burns and Corwin, 2013; Takeda et al., 2018). However, our understanding of the molecular mechanisms that regulates supporting cell behavior is limited. Our results indicate that DUSP14 may be an important regulator of supporting cell development. Furthermore, *DUSP14* is also reported to be involved in the hepatocyte proliferation and regeneration (Wang X. et al., 2018). In the immune system, DUSP14 negatively regulates β cell proliferation and apoptosis of cancer cells (Klinger et al., 2008).

Here, we explore the molecular mechanisms that lead to hair cell regeneration in zebrafish by exploiting their ability to regenerate hair cells. To our knowledge, this study reported the important effects of *dusp14* gene on the fate of the hair cell in zebrafish for the first time, mainly through regulating proliferation of supporting cells, providing a new insight into understand the mechanism of supporting cell development and a new potential target for the treatment of hearing loss.

## Data Availability Statement

The data presented in this study are deposited in the China National GeneBank DataBase (CNGBdb), accession number: CNP0002521 (https://db.cngb.org/search/project/CNP0002521/).

## Ethics Statement

The animal study was reviewed and approved by Animal Care and Use Committee of Nantong University.

## Author Contributions

DL and FC supervised and designed this project. GW, CC, XZ, JS, and FQ wrote the manuscript. GW, XZ, CG, and FQ analyzed the data. XZ, MX, CW, and QG performed the experiments. All authors contributed to the article and approved the submitted version.

## Conflict of Interest

The authors declare that the research was conducted in the absence of any commercial or financial relationships that could be construed as a potential conflict of interest.

## Publisher’s Note

All claims expressed in this article are solely those of the authors and do not necessarily represent those of their affiliated organizations, or those of the publisher, the editors and the reviewers. Any product that may be evaluated in this article, or claim that may be made by its manufacturer, is not guaranteed or endorsed by the publisher.

## Supplementary Material

Supplementary Figure 1 The design of *Dusp14*-Mo sequence and verification of the knockdown efficiency. **(A)** The design of *dusp14* morphant sequence. **(B)** Representative images of Dusp14^+^ cells of neuromasts in the posterior lateral line of the control and *dusp14* morphants. **(B′)** The statistical results of panel **(B)**. **(C)** *Left*: the result of *ef1*α RT-PCR of wild-type zebrafish and *dusp14* morphants. *Right*: the result of *dusp14* RT-PCR of wild-type zebrafish and *dusp14* morphants. **(C′)** The statistical results of panel **(C)**.

Supplementary Figure 2 *Dusp14* knockout leads to a decrease in zebrafish hair cell. **(A)** The design of *dusp14* guide-RNA. **(B)** Mutations occurred in the target site of the *dusp14* gene in mutant zebrafish compared to the wild-type fish. **(C)** Confocal imaging analysis of the numbers of hair cell clusters and hair cells in the lateral line system of wild-type and *dusp14* mutants at 72 hpf. **(C′)** The statistical analysis of panel **(C)**. Experimental embryos were sampled at 72 hpf (*n* > 7). Each bar represents the mean ± SEM. Values with *, **, ***, and **** above the bars are significantly different (*p* < 0.05, *p* < 0.01, *p* < 0.001, and *p* < 0.0001, respectively).

Supplementary Figure 3 *Dusp14* knockdown did not induce zebrafish hair cell apoptosis. **(A)** DAPI and cleaved TUNEL staining for the L3 hair cell clusters in the posterior lateral line of the control zebrafish and *dusp14* morphants. **(B)** DAPI and cleaved caspase-3 staining for the L3 hair cell clusters in the posterior lateral line of the control zebrafish and *dusp14* morphants.

Supplementary Figure 4 qRT-PCR of different expression gene in the control and *dusp14* morphants. **(A)** Twelve DEGs were detected in transcriptome sequencing. **(B)** The results of 12 DEGs caused by *dusp14* morphants at 72 hpf (*n* = 3). Each bar represents the mean ± SEM. Values with *, **, ***, and **** above the bars are significantly different (*p* < 0.05, *p* < 0.01, *p* < 0.001, and *p* < 0.0001, respectively).

Supplementary Table 1 Transcriptomic profiling of *dusp14*-morphant and wild-type zebrafish. The number of reads and percentage mapped to the coding genes of zebrafish genome is calculated. The 90% mapped rate presents the proportion of reads matched to the coding genes of zebrafish genome in the totally valid reads.

Supplementary Table 2 Differentially expressed genes obtained based on transcriptome sequencing.

Supplementary Table 3 Primer information.
