# Supplementary figures and images for: Dual-Specificity Phosphatase 14 Regulates Zebrafish Hair Cell Formation Through Activation of p38 Signaling Pathway
Source: Front Cell Neurosci. 2022 Mar 23;16:840143. doi: 10.3389/fncel.2022.840143 (PMC8984152; doi:10.3389/fncel.2022.840143)

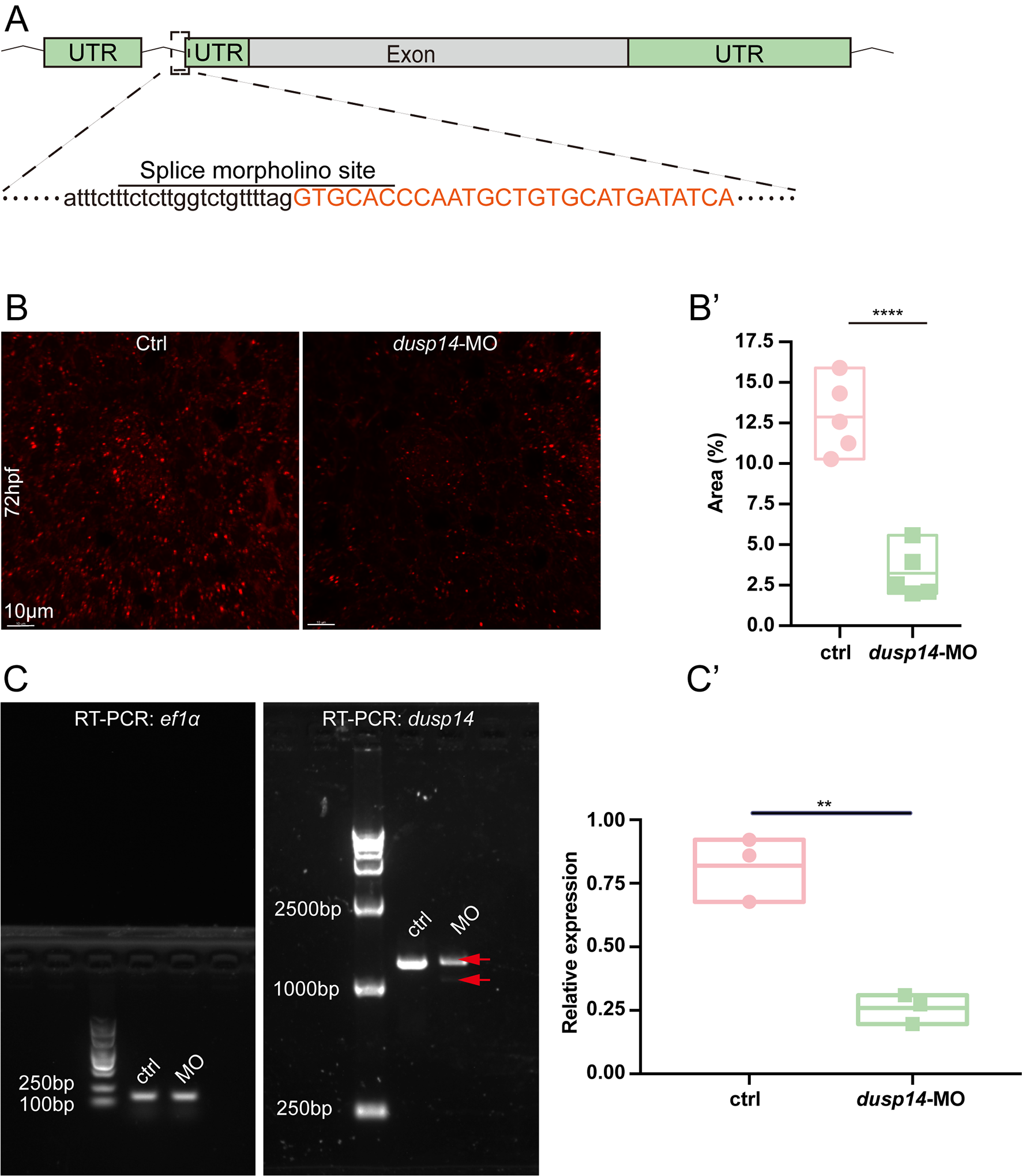

Supplement: Supplementary Figure 1 — The design of Dusp14-Mo sequence and verification of the knockdown efficiency. (A) The design of dusp14 morphant sequence. (B) Representative images of Dusp14+ cells of neuromasts in the posterior lateral line of the control and dusp14 morphants. (B′) The statistical results of panel (B). (C) Left: the result of ef1α RT-PCR of wild-type zebrafish and dusp14 morphants. Right: the result of dusp14 RT-PCR of wild-type zebrafish and dusp14 morphants. (C′) The statistical results of panel (C). [file Image_1.TIF]

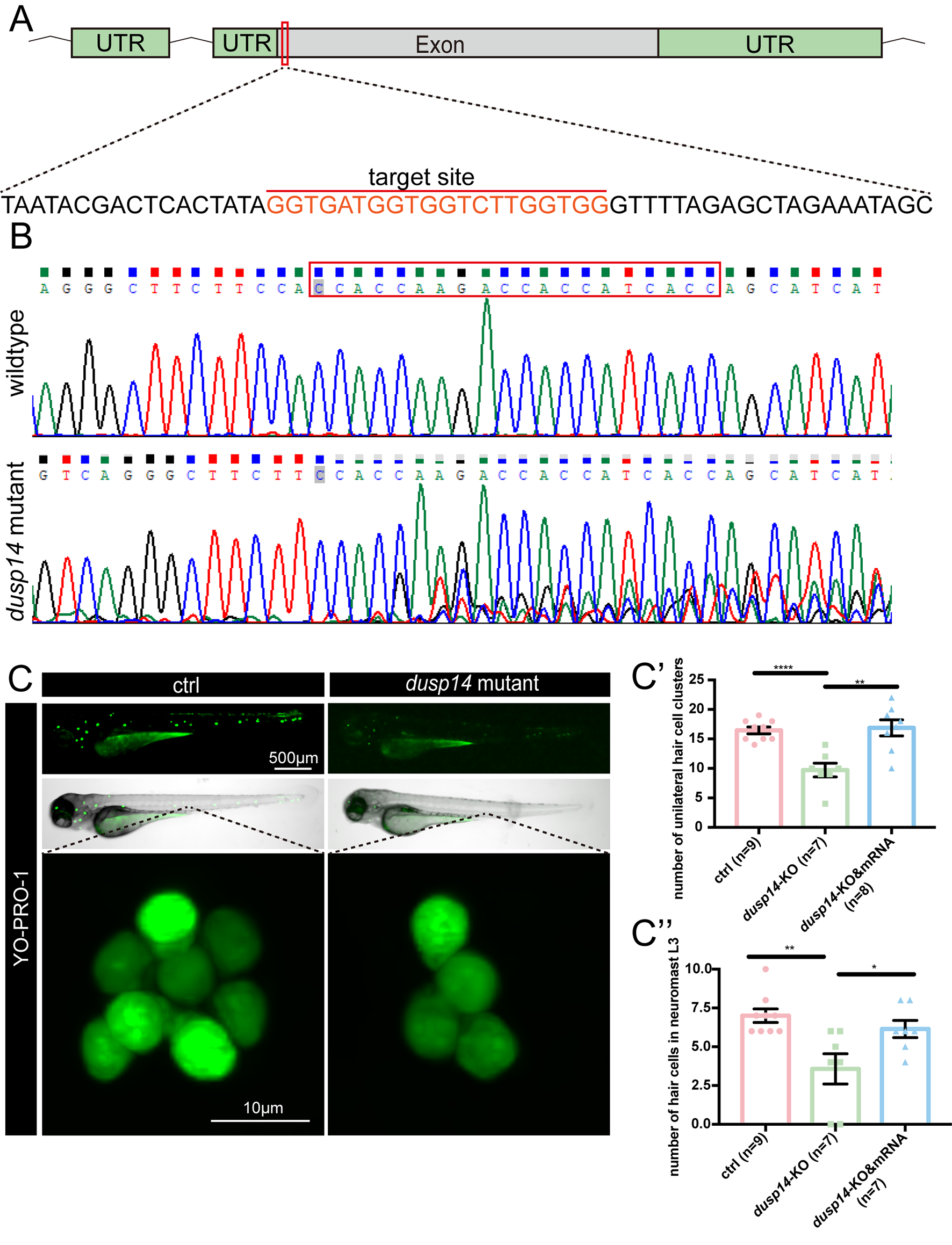

Supplement: Supplementary Figure 2 — Dusp14 knockout leads to a decrease in zebrafish hair cell. (A) The design of dusp14 guide-RNA. (B) Mutations occurred in the target site of the dusp14 gene in mutant zebrafish compared to the wild-type fish. (C) Confocal imaging analysis of the numbers of hair cell clusters and hair cells in the lateral line system of wild-type and dusp14 mutants at 72 hpf. (C′) The statistical analysis of panel (C). Experimental embryos were sampled at 72 hpf (n > 7). Each bar represents the mean ± SEM. Values with *, **, ***, and **** above the bars are significantly different (p < 0.05, p < 0.01, p < 0.001, and p < 0.0001, respectively). [file Image_2.TIF]

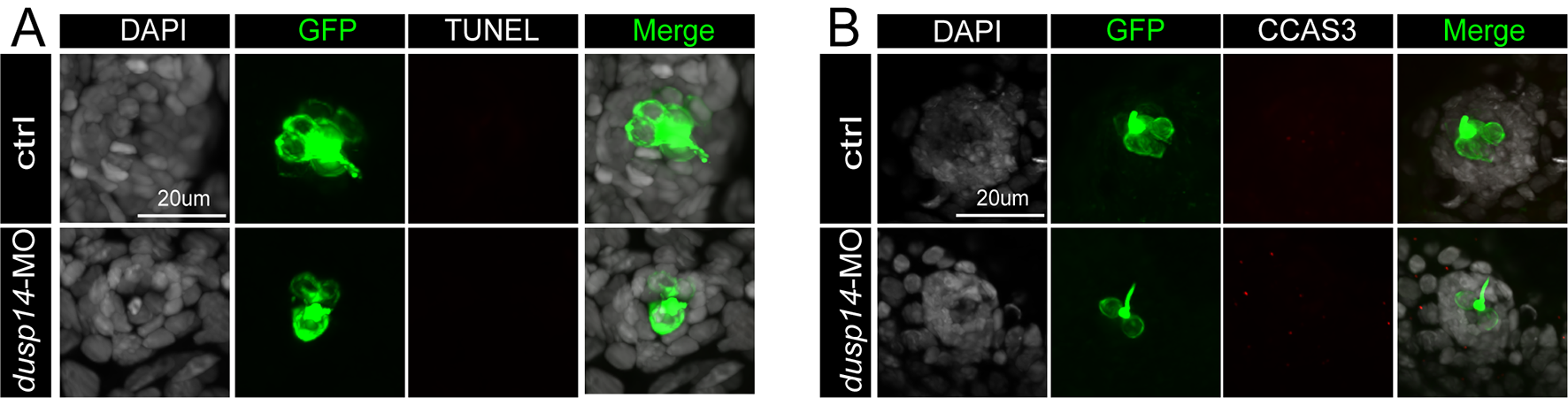

Supplement: Supplementary Figure 3 — Dusp14 knockdown did not induce zebrafish hair cell apoptosis. (A) DAPI and cleaved TUNEL staining for the L3 hair cell clusters in the posterior lateral line of the control zebrafish and dusp14 morphants. (B) DAPI and cleaved caspase-3 staining for the L3 hair cell clusters in the posterior lateral line of the control zebrafish and dusp14 morphants. [file Image_3.TIF]

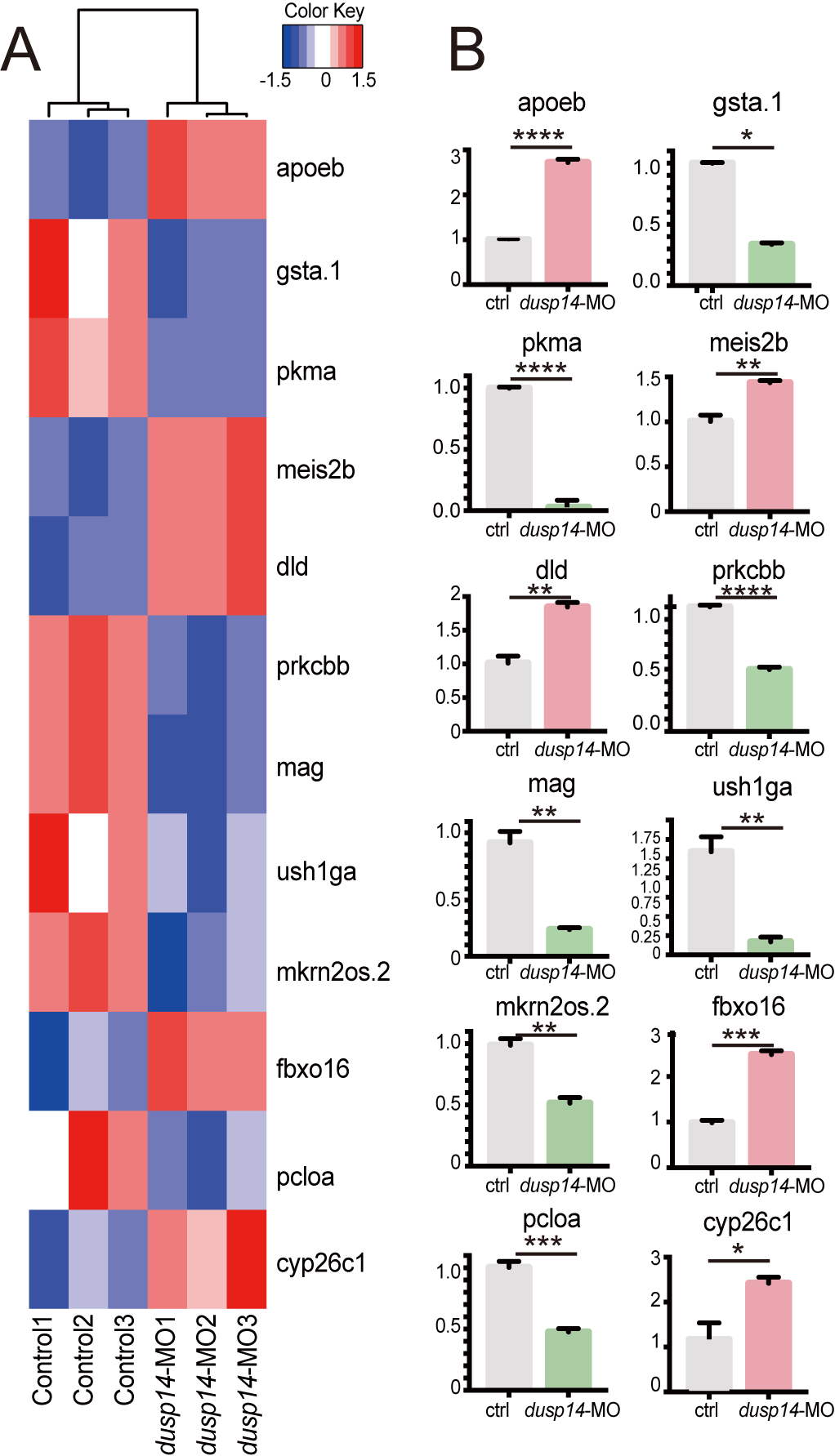

Supplement: Supplementary Figure 4 — qRT-PCR of different expression gene in the control and dusp14 morphants. (A) Twelve DEGs were detected in transcriptome sequencing. (B) The results of 12 DEGs caused by dusp14 morphants at 72 hpf (n = 3). Each bar represents the mean ± SEM. Values with *, **, ***, and **** above the bars are significantly different (p < 0.05, p < 0.01, p < 0.001, and p < 0.0001, respectively). [file Image_4.TIF]
