# Supplementary material for: Dual-Specificity Phosphatase 14 Regulates Zebrafish Hair Cell Formation Through Activation of p38 Signaling Pathway
Source: Front Cell Neurosci. 2022 Mar 23;16:840143. doi: 10.3389/fncel.2022.840143 (PMC8984152; doi:10.3389/fncel.2022.840143)
Supplement: Supplementary Table 1 — Transcriptomic profiling of dusp14-morphant and wild-type zebrafish. The number of reads and percentage mapped to the coding genes of zebrafish genome is calculated. The 90% mapped rate presents the proportion of reads matched to the coding genes of zebrafish genome in the totally valid reads. [file Data_Sheet_1.PDF]

STable 1 Transcriptomic Profiling of Dusp14-morphant and Wild-Type Zebrafish.

The number of reads and percentage mapped to the coding genes of Zebrafish genome are calculated. The % mapped rate presents the proportion of reads matched to the coding genes of Zebrafish genome in the totally valid reads.

Samples	Total reads	Total mapped	%Mapped
control 1	48174784	44324630	92.01%
control 2	42054838	38112740	90.63%
control 3	39336634	35663882	90.66%
dusp14 1	49443368	44626894	90.26%
dusp14 2	48680900	43896677	90.17%
dusp14 3	41810874	37562713	89.84%
